# Food safety knowledge, attitude, and hygiene practices of street-cooked food handlers in North Dayi District, Ghana

**DOI:** 10.1186/s12199-021-00975-9

**Published:** 2021-05-03

**Authors:** Lawrence Sena Tuglo, Percival Delali Agordoh, David Tekpor, Zhongqin Pan, Gabriel Agbanyo, Minjie Chu

**Affiliations:** 1 Department of Epidemiology, School of Public Health, Nantong University, 9 Seyuan Road, Nantong, Jiangsu China; 2 Department of Nutrition and Dietetics, School of Allied Health Sciences, University of Health and Allied Sciences, Ho, Ghana; 3 North Dayi District Health Directorate, Volta Region, Ghana Health Service, Accra, Ghana

**Keywords:** Food safety, Knowledge, Attitude, Hygiene practice, Street-cooked food handlers

## Abstract

**Background:**

Food safety and hygiene are currently a global health apprehension especially in unindustrialized countries as a result of increasing food-borne diseases (FBDs) and accompanying deaths. This study aimed at assessing knowledge, attitude, and hygiene practices (KAP) of food safety among street-cooked food handlers (SCFHs) in North Dayi District, Ghana.

**Methods:**

This was a descriptive cross-sectional study conducted on 407 SCFHs in North Dayi District, Ghana. The World Health Organization’s Five Keys to Safer Food for food handlers and a pretested structured questionnaire were adapted for data collection among stationary SCFHs along principal streets. Significant parameters such as educational status, average monthly income, registered SCFHs, and food safety training course were used in bivariate and multivariate logistic regression models to calculate the power of the relationships observed.

**Results:**

The majority 84.3% of SCFHs were female and 56.0% had not attended a food safety training course. This study showed that 67.3%, 58.2%, and 62.9% of SCFHs had good levels of KAP of food safety, respectively. About 87.2% showed a good attitude of separating uncooked and prepared meal before storage. Good knowledge of food safety was 2 times higher among registered SCFHs compared to unregistered [cOR=1.64, *p*=0.032]. SCFHs with secondary education were 4 times good at hygiene practices of food safety likened to no education [aOR=4.06, *p*=0.003]. Above GHc1500 average monthly income earners were 5 times good at hygiene practices of food safety compared to below GHc500 [aOR=4.89, *p*=0.006]. Registered SCFHs were 8 times good at hygiene practice of food safety compared to unregistered [aOR=7.50, *p*<0.001]. The odd for good hygiene practice of food safety was 6 times found among SCFHs who had training on food safety courses likened to those who had not [aOR=5.97, *p*<0.001].

**Conclusions:**

Over half of the SCFHs had good levels of KAP of food safety. Registering as SCFH was significantly associated with good knowledge and hygiene practices of food safety. Therefore, our results may present an imperative foundation for design to increase food safety and hygiene practice in the district, region, and beyond.

**Supplementary Information:**

The online version contains supplementary material available at 10.1186/s12199-021-00975-9.

## Introduction

A report by the World Health Organization (WHO) (2015) showed that about two million incurable cases of food poisoning materialize annually in unindustrialized nations. The WHO further estimates that 600 million food-borne diseases (FBDs) each year were related to poor food safety and hygiene practice with 420,000 deaths [1], the majority attributed to meat-related vulnerabilities [2]. About, 76 million FBDs caused 325,000 hospitalizations in the USA which led to 5000 deaths [3]. The source was associated with the consumption of turkey contaminated by *Salmonella enterica serovar Heidelberg*, responsible for salmonellosis in the USA [4]. Almost, 1.3 million FBDs resulted in 21,000 hospital stays reported in England which led to 500 deaths. The contamination was due to sprouts by *Escherichia coli O104* [3]. Around 53% of the food-borne problems and 31% of its associated illness were attributed to meat consumption in the Netherlands [2]. The rate of FBDs in Malaysia was 47.8% out of 100,000 people who patronized street-cooked foods [5]. In Ghana, about 65,000 persons including 5000 kids below 5 years died yearly due to FBDs [6].

The risk factors such as inappropriate time interval, unsuitable temperature, weather condition, unhygienic activities, unacceptable handling of foods, foodstuff from insecure origins, impoverished self-cleanliness, improper cleaning of cooking materials, using untreated water, and improper food storages were attributed to the causes of FBDs [7–9]. Also, neglect of hygienic measures by food handlers has been implicated as enablers for the spread of pathogenic microorganisms [10] and the cause of infections among consumers [11].

Studies recount that 12 to 18% of food-borne illnesses are attributable to contaminations [12, 13], poor food safety, and inappropriate hygiene practices which were accredited to street-cooked food handlers (SCFHs) [14, 15]. These SCFHs are people who are wholly or partly engaged in the food preparation, processing, and production value chain and who have a direct touch on food and cooking utensils [9, 16]. Foods prepared by food handlers under unhygienic conditions become a public health concern both in industrialized and low-income countries [17]. Food safety and hygienic practices of SCFHs are essential to ensure that food is free from any forms of contamination through preparation and processing for consumption and to prevent the spread of FBDs [18, 19].

Food safety knowledge (FSK) is the understanding of food learned from skills or schooling, food safety attitude (FSA) refers to sensation or belief about food safety, and food safety practice refers (FSP) to the act or use of food safety [20]. Food safety knowledge, attitude, and practices (KAP) are important because inadequate knowledge, poor attitude, and poor sanitation practices by SCFHs have a severe danger to food safety applications in food companies [21]; hence, KAP of food safety contributes significantly to the occurrence of food poisoning and FBDs among consumers [22].

A study conducted in Brazil among food truck food handlers revealed poor hygiene, poor clean observes, poor environments, and higher contaminated meals [23]. The problem of FBDs was higher in Southeast Asian and African counties [24]. Ma et al. [25] study in China, among street food vendors, revealed poor behaviour practices and knowledge of food safety among the respondents. Tabit and Teffo [26] in South Africa found over 60% of the respondents keep good knowledge and acceptable hygiene performance of food safety. Lema et al. [27] in Ethiopia reported that below half of the respondents obtained good food cleanliness applications. The effects of food-related illness expenditures in hospital treatments are about US$ 110 billion annually in developing countries, which resulted in decreasing production [28].

The recurrent happenings of food-related illnesses brought in its wake concerns about the food safety knowledge and hygiene among SCFHs [29]. Maintaining food safety involves establishing global laws conferring to an agreement between institutions that actualized this agenda [30, 31]. The Government of Ghana affirmed food safety regulations in collaboration with the Food and Drug Authority (FDA) [30]. Yet, its application is undermined due to ineffective supervision by appropriate agencies [32]. The problem was due to the broad governmental assembly in cities and communities under the local administration [31]. Some local studies conducted in the four regions of Ghana such as Greater Accra, Northern, Western, and Central have reported adequate knowledge, good attitude, and positive behavioural practices of food safety and handling practices [11, 33–35]. Studies have shown that SCFHs were not knowledgeable about the WHO’s Five Keys to Safer Food for food handlers [33, 36] which include keeping clean, separating raw and cooked food, cooking thoroughly, keeping food at safe temperatures, and using safe water and raw materials [37].

Hence, the acceptance and the use of the KAP instrument as a problem-solving approach in this study are validated from previous researches [23, 38, 39]. This would adequately support the policymaking development and the change of embattled intervention policies for the prevention and control of FBDs. The KAP’s tool assessment defined in this study is considered appropriate to other frameworks if the statements in the KAP’s sections are validated. To our knowledge, no research has yet been done on KAP of food safety among SCFHs selling commonly consumable foods on the street in Volta Region, Ghana. Hitherto, the high cases of FBDs such as diarrhoea, cholera, and typhoid fever outbreak occurrences in the district are presumed to be influenced by SCFHs. The KAP of SCFHs on food safety and hygiene precautions ruins uncertainty in the district, and a swift policy to mend some causes central to the occurrence of FBDs is obligatory. This would help the District Health Directorate’s regulatory agency to plan the prevention methods. Therefore, this study assessed knowledge, attitude, and hygiene practices of food safety on SCFHs in North Dayi District, Ghana.

## Materials and methods

### Study design and setting

This study was a descriptive cross-sectional carried out between August and November 2020 and used a validated, pretested, and structured questionnaire to collect data from stationary SCFHs along the principal streets within North Dayi District. North Dayi District is one of the 18 administrative districts in the Volta Region, Ghana [40]. It shares boundaries with Kpando Municipal to the north, South Dayi District to the south, and Afadzato South District to the east. The entire residents of the North Dayi District are 39,913 covering 46.7% men and 53.3% women [40]. The people of the District constitute 1.9% of the total population of the Volta Region [40]. Farming is the foremost financial activity, making it one of the main sources of income in the district [40]. We carried out this study because of the recent cases of food-borne illness reported among the residents such as diarrhoea, cholera, and typhoid fever in the district [41].

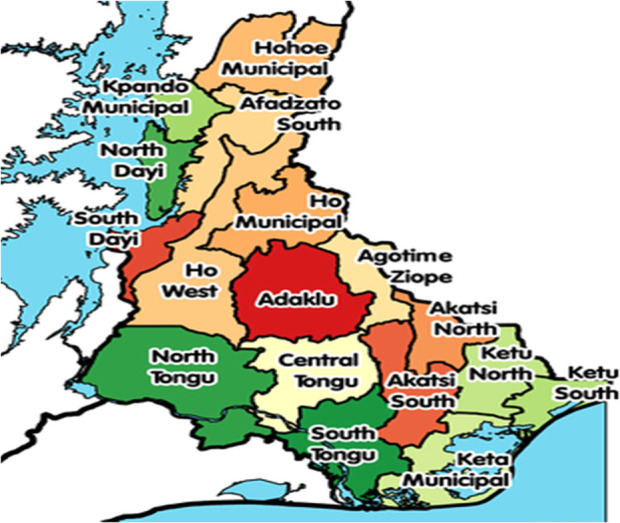



### Eligibility criteria

Stationary SCFHs who directly served already cooked food to customers and those who owned their outlets were included in the study. SCFHs who dissented to partake in the research were excepted including all assistants and helpers. The assistants and helpers were excluded because not all vendors had assistants or helpers and they tend to be more in numbers than the vendor-owners themselves. So for as not to allow bias in the results, we chose to sample only the vendor-owners. Moreover, vendor-owners tend to have direct responsibility for monitoring the food safety environment of their vending sites; hence, we chose to sample them as the focus of this study.

### Sample size and sampling

Cochran’s formula *Z*^2^*p* (1 − *p*)/*e*^2^ [42] for unknown study populations was used. Since a similar study in the Volta Region of Ghana among the population subgroup is unavailable, 50% was used for response distribution, with 95% confidence level, and a margin of error of 5% for the populace, plus 10% non-response rate which gave us a sample size of 423.

### Data collection tools

A structured questionnaire was designed based on different studies conducted globally [16, 20, 38, 39, 43–46]. Similar versions of the questionnaires were used in studies conducted in Ghana [47–49]. The instrument was distributed into 4 parts: socio-demographics, knowledge, attitude, and hygiene practices. The statements on KAP were adapted from the WHO’s Five Keys to Safer Food guidebook for food handlers [37]. The questionnaire was firstly designed in English, then converted to local dialects, and translated back to English to ensure reliability and simplicity of the question. Four professionals in the field of the study assessed the face and the content validity of the questionnaire. The questionnaire was pretested on 12 stationary SCFHs in Tanyigbe located 7 km from the study area. The pretesting findings were not added to the main study but were used to modify some questions to improve their clarity. The most pertinent modifications done on the study instrument were a cooked meal should stay hot more than 60°C before serving, putting uncooked and prepared meal separating prevent cross-contamination, and checking and dispose of meal that past their expiry date. The data were collected through trained research assistant-led interviews which lasted for about 25 min per respondent. The interviewer-administered questionnaire was given to the SCFHs who could read and write to answer by themselves while those SCFHs who could not read and write have been aided by the research assistants in answering the questionnaire.

#### Determination of knowledge, attitude, and hygiene practices on food safety

##### Knowledge

Section 2 of the questionnaire contained 10 structured questions on knowledge of food safety with 3 likely responses; “true”, “false”, and “do not know”. The questions precisely covered the respondents’ knowledge of individual cleanliness, food-borne illnesses, microbes, infection control, and sanitary practices. Each correct knowledge item reported was awarded a score of 1 point. Incorrect knowledge was awarded a 0 score (including “do not know”). In this study, if “true” is the correct answer, then “true” is score 1 point while “false” is score 0 point or otherwise reverse.

##### Attitude

Queries relating to attitudes in the third segment of the questionnaire were designed to assess the knowledge of SCFHs on food wellbeing and hygiene. This part of the section assessed psychological state concerning views, opinion, morals, and characters to act in particular [21, 48]. It contains 10 structured queries with 3 likely answers: “agree”, “disagree”, and “not sure”. Each correct attitude reported was awarded a score of 1 point while the other incorrect attitude option was rated a 0 score (including “not sure”). In this study, if “agree” is the correct answer, then “agree” is score 1 point while “disagree” is score 0 point or otherwise reverse.

##### Hygiene practice

Section 4 of the questionnaire measured food hygiene and sanitation practices of SCFHs. It contained 10 structured queries with 2 likely answers: “yes” and “no”. Each correct hygiene practice reported was awarded a score of 1 point while incorrect hygiene practices reported were awarded a score of 0. This method of assessment was used in previous studies [28]. In this study, if “yes” is the correct answer, then “yes” is score 1 point while “no” is score 0 point or otherwise reverse.

The grouping method is appropriate and suitable for studies allied to the assessment “of food handlers” KAP of food safety and hygiene [27, 28, 34, 46, 47, 50–52]. The knowledge and attitude questions with “do not know” or “not sure”, thus the third option, had been presented to enable simplicity of responding by SCFHs for fascinating for thoughts considered by an undecided or doubtfulness [28]. This third option “do not know” or “not sure” always scores a 0 point due to the cumulative percentage approach adapted which considers only the acceptable response or the correct answer [53]. The cumulative percentage scoring method of assessment considers only the acceptable answer and the total cumulative score is converted to 100% [53]. The cumulative scores below 70% of the acceptable responses on WHO’s Five Keys to Safer Food-related knowledge, attitude, and hygiene practices were considered as “poor”, and cumulative scores 70% and higher were considered as “good” [27, 34, 39, 46, 48].

### Data analysis

Questionnaires were checked manually before entering into Microsoft Excel 2016 spreadsheet. Coding and analysis were done in IBM Statistical Package for Social Sciences (SPSS Inc., Chicago, USA; https://www.spss.com) version 24.0. Categorical variables were expressed as frequency and percentage. The disparity between categorical variable groups was verified using the Fisher exact or chi-square test where appropriate. Significant parameters were used in bivariate and multivariate logistic regression models to calculate the power of the relationships observed. A *p*-value <0.05 was considered statistically significant.

### Ethical consideration

Approval was sought from Ghana Health Service, North Dayi District Health Directorate, with the identity (NDDHD/GR/002/20) 15/07/2020. The research assistants introduced themselves and written informed permission was sought from the respondents. The research method was plainly explained to the respondents in their native dialects (English, Ewe, or Twi). Participants were identified by study numbers. The study numbers of the participants were kept in both locked files and secured computer files and accessible only to key investigators. All data were anonymized and unlinked to the respondents’ identities during the data analysis.

## Results

### Demographic data

**Fig. 1:**
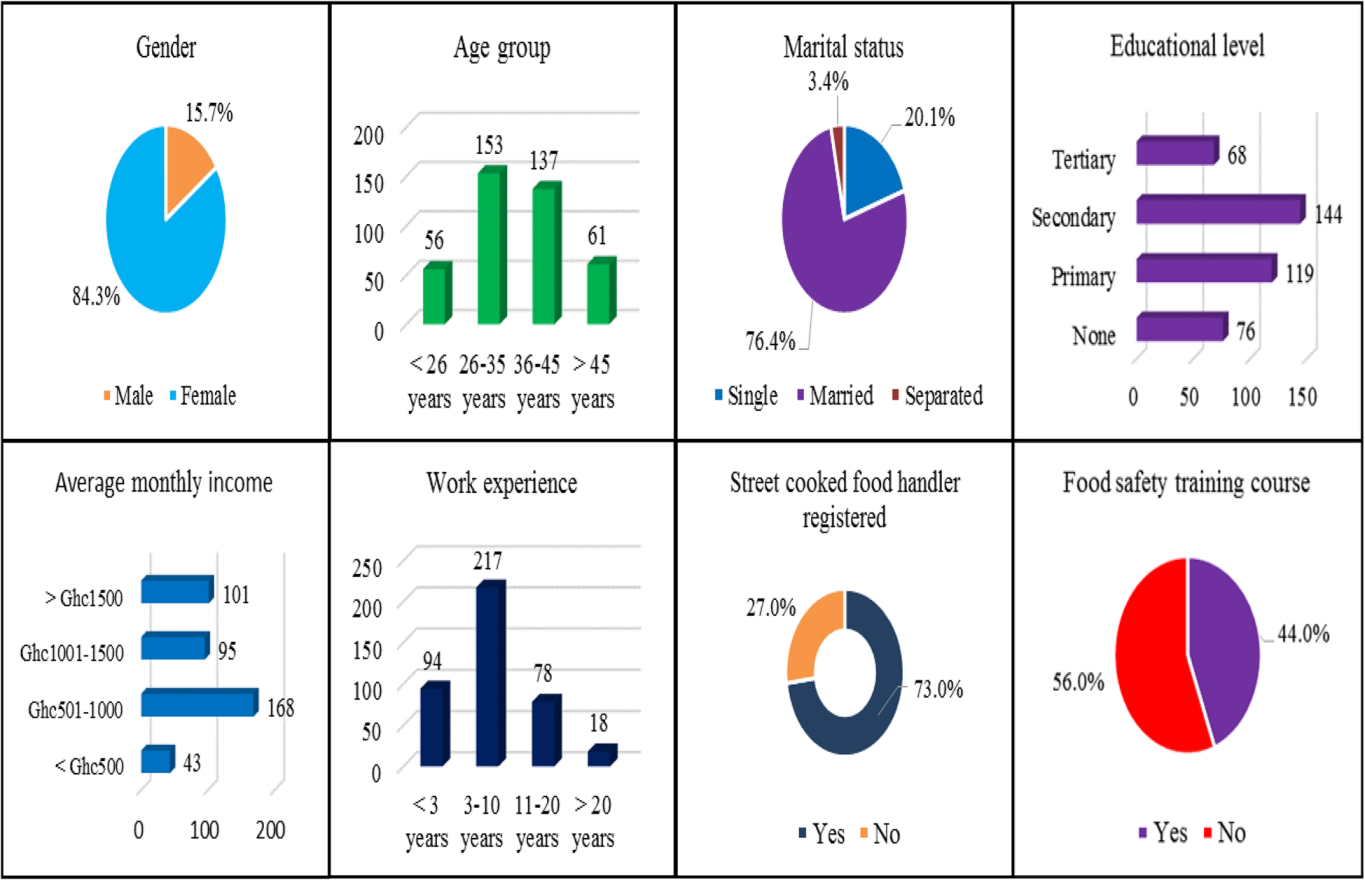
Demographic data of respondents

A total complete of 423 questionnaires were conveniently distributed for data collection based on the availability of SCFHs at their dedicated vending sites. Questionnaires of 407 were fully answered and collected from the respondents with a 96.2% (407/423) success rate. *n*=*Z*^2^*p* (1 − *p*)/*e*^2^  = 1.96^2^0.5 (1 − 0.5)/0.05^2^ =384.16+38.416 =422.576. The majority (*n*=343; 84.3%) of SCFHs were female, were between the age range of 26 and 35 years (*n*=153; 37.6%), and were married (*n*=311; 76.4%). Over one-third (*n*=144; 35.4%) of SCFHs had attained secondary education. Most (*n*=168; 41.3%) of SCFHs earned an average monthly income between GHc501 and GHc1000. Over half (*n*=217; 53.3%) of SCFHs had 3–10 years of working experience. Regarding SCFH registered, *n*=297 (73.0%) reported that they have registered. More than half (*n*=228; 56.0%) of SCFHs had not attended a food safety training course (Fig. 1).

### Food safety knowledge

Almost all (*n* = 381; 93.6%) of SCFHs knew about the washing of hands for 1 min using water and soap before touching food. The majority (*n*=313; 76.9%) of SCFHs knew that similar chopping board should not be used for uncooked and prepared foods if it appears wash; *n* = 336 (82.6%) knew that cooked meal should stay hot before serving (more than 60°C); and *n* = 275 (67.6%) knew that excess meal should be kept at zone temperature and eat for the following mealtime. Most (*n*=239; 58.7%) of SCFHs knew that uncooked meal should be kept individually from a prepared meal; *n* = 363 (89.2%) knew that treated water should be used for cooking; *n* = 363 (89.2%) knew that cockroach and house flies should not be allowed into the kitchen; and *n* = 274 (67.3%) knew that wiping cloths can spread microorganisms and cause disease. However, the majority (*n*=235; 57.7%) of SCFHs did not know that food cooking utensils should not be cleaned using tap water only. Also, *n* = 202 (49.6%) of SCFHs did not know that fresh meat should not be stowed anyplace in the fridge once it is cool (Table 1).

**Table 1:** Food safety knowledge of the respondents (*n*=407)

Knowledge questions	Responses *n* (%)		
	True	False	Do not know
Wash hands for 1 min using water and soap before touching food	381 (93.6)	11 (2.7)	15 (3.7)
A similar chopping board should be used for uncooked and prepared foods if it appears to wash	32 (7.9)	313 (76.9)	62 (15.2)
A cooked meal should stay hot before serving (more than 60°C)	336 (82.6)	47 (11.5)	24 (5.9)
An excess meal should be kept at zone temperature and eat for the following mealtime	91 (22.4)	275 (67.6)	41 (10.1)
Food cooking utensils should be cleaned using tap water only	235 (57.7)	157 (38.6)	15 (3.7)
An uncooked meal should be kept individually from the prepared meal	239 (58.7)	111 (27.3)	57 (14.0)
Fresh meat should be stowed anyplace in the fridge once it is cool	202 (49.6)	165 (40.5)	40 (9.8)
Treated water should be used for cooking	363 (89.2)	35 (8.6)	9 (2.2)
Cockroach and house flies should be allowed into the kitchen	32 (7.9)	363 (89.2)	12 (2.9)
Wiping cloths can spread microorganisms and cause disease	274 (67.3)	59 (14.5)	74 (18.2)

### Food safety attitude

The majority (*n*=277; 68.1%) of SCFHs disagreed that regular hand cleaning throughout meal processing is needless; *n* = 323 (79.4%) agreed that cleaning kitchen shells lessen the danger of infection, and *n* = 355 (87.2%) agreed that putting uncooked and prepared meal separating stop infection. Below half (*n*=181; 44.5%) of SCFHs agreed that they should be able to differentiate healthy diets and rotten food through eyeing; *n*=262 (64.4%) disagreed that using different knives and chopping materials for a fresh and prepared meal require more time; *n* = 366 (89.9%) agreed that they cough or sneeze inside the elbow if towel or paper not available; *n* = 291 (71.5%) agreed that checking meal for cleanliness and healthiness is important; and *n*=377 (92.6%) agreed that it is vital to dispose of meals that have gotten to expiring date. Nevertheless, *n* = 332 (81.6%) of SCFHs agreed that it is acceptable to use the same cloth for dusting and drying and *n*=217 (53.3%) disagreed that is unhealthy to allow prepared meal stay outside of the fridge for over 2 h (Table 2).

**Table 2:** Food safety attitude of the respondents (*n*=407)

Attitude questions	Agree	Disagree	Not sure
	No. (%)	No. (%)	No. (%)
Regular hand cleaning throughout meal processing is needless	114 (28.0)	277 (68.1)	16 (3.9)
Cleaning kitchen shells lessens the danger of infection	323 (79.4)	75 (18.4)	9 (2.2)
Putting uncooked and prepared meal separating stop infection	355 (87.2)	27 (6.6)	25 (6.1)
Acceptable to use the same cloth for dusting and drying	332 (81.6)	41 (10.1)	34 (8.4)
Can differentiate healthy diets and rotten food by eyeing	181 (44.5)	156 (38.3)	70 (17.2)
Is unhealthy to allow prepared meal stay outside of the fridge for over 2 h	139 (34.2)	217 (53.3)	51 (12.5)
Using different knives and chopping materials for a fresh and prepared meal need more time	123 (30.2)	262 (64.4)	22 (5.4)
Cough or sneeze inside the elbow if towel or paper not available	366 (89.9)	31 (7.6)	10 (2.5)
Checking meal for cleanliness and healthiness is important	291 (71.5)	81 (19.9)	35 (8.6)
It is vital to dispose of meals that have gotten to the expiring date	377 (92.6)	24 (5.9)	6 (1.5)

### Food safety hygiene practice

The majority (*n*=343; 84.3%) of SCFHs cleaned their fingers throughout meal cooking; *n* = 267 (65.6%) washed their cooking utensils used to cook a meal before using for a different meal; *n*=234 (57.5%) used different cooking bowls and chopping material if cooking a fresh and prepared meal; and *n*=359 (88.2%) dispersed uncooked and prepared meal before preservation. Also, *n*=278 (68.3%) keep prepared food at room temperature for 2 h when finished cooking; *n*=269 (66.1%) checked and disposed of meal past its expiry date; *n*=372 (91.4%) cleaned fresh food that needs no cooking before consumption; *n*=320 (78.6%) inspected if a meal is cooked by eyeing; and *n*=359 (88.2%) examined if a meal is grilled by touching it. Moreover, *n*=253 (62.2%) used similar kitchen cloth to clean shells and hands (Table 3).

**Table 3:** Food safety hygiene practice of the respondents (*n*=407)

Hygiene practice questions	Responses *n* (%)	
	Yes	No
Clean your fingers throughout meal cooking	343 (84.3)	64 (15.7)
Wash cooking utensils used to cook a meal before using for different meal	267 (65.6)	140 (34.4)
Use different cooking bowls and chopping material if cooking a fresh and prepared meal	234 (57.5)	173 (42.5)
Dispersed uncooked and prepared meal before preservation	359 (88.2)	48 (11.8)
Keep prepared food at room temperature for 2 h when finished cooking	278 (68.3)	129 (31.7)
Check and disposed of meal past their expiry date	269 (66.1)	138 (33.9)
Clean fresh food that needs no cooking before consumption	372 (91.4)	35 (8.6)
Inspect if a meal is cooked by eyeing	320 (78.6)	87 (21.4)
Use similar kitchen cloth to clean shells and hands	253 (62.2)	154 (37.8)
Examine if a meal is grilled by touching it	359 (88.2)	48 (11.8)

### SCFH knowledge, attitude, and hygiene practice on food safety classification

**Fig. 2:**
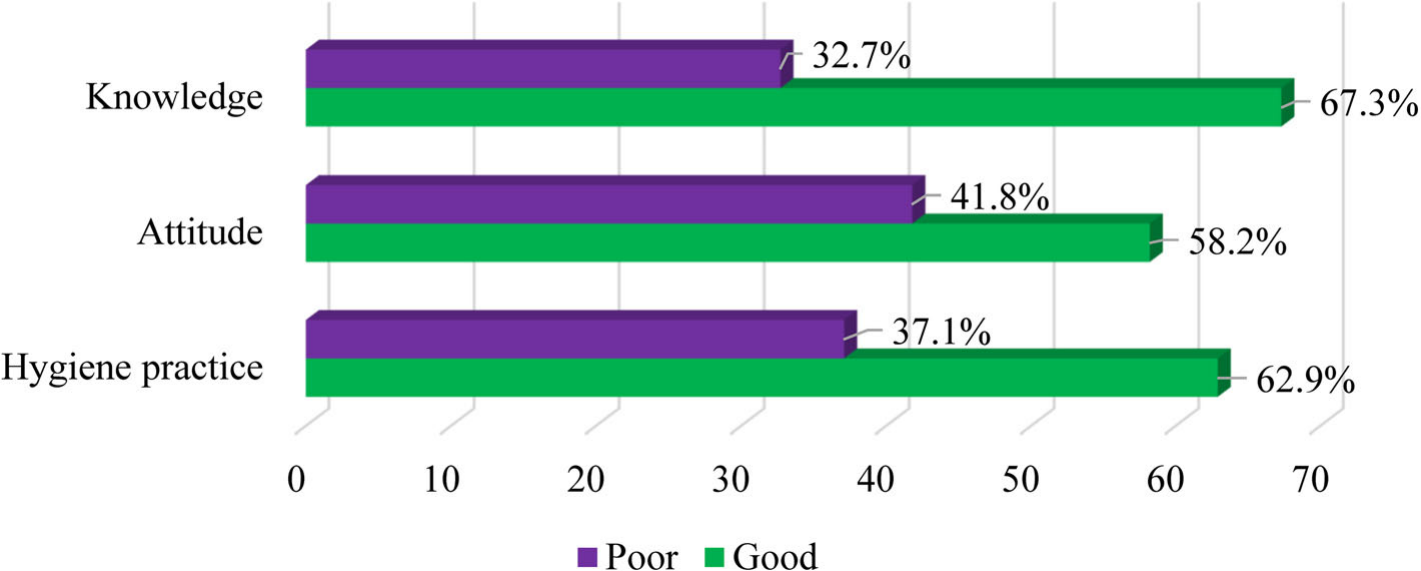
Levels of respondents’ knowledge, attitude, and hygiene practice on food safety

A high proportion (*n*=274, 67.3%; *n*=237, 58.2%; and *n*=256, 62.9%) of SCFHs had good levels in knowledge, attitude, and hygiene practices on food safety (Fig. 2).

#### Association between knowledge, attitude, and hygiene practice and demographic data

Statistical significance was observed in the knowledge section among registered SCFHs (*p*=0.031). None of the respondent’s socio-demographic data was statistically significant in the attitude section of food safety *p* < 0.05. The study found significant differences (*p*<0.05) in the hygiene practice scores section with the educational status, average monthly income, registered SCFHs, and SCFHs completing food safety training course of food safety among SCFHs (Table 4). The odds ratio showed registered SCFHs were 1.6 times good at food safety knowledge likened to unregistered SCFHs [cOR=1.64 (95% CI 1.04–2.59), *p*=0.032]. The logistic regression analysis revealed that respondents who had secondary education were 4.1 times good at hygiene practice of food safety [aOR=4.06 (95% CI 1.63–10.11), *p*=0.003] compared to informal education. The respondents with average monthly income greater than GHc1500 were 4.9 times more likely to have good food safety and hygiene practices compared to those who earned less than Ghc500 average monthly income [aOR=4.89 (95% CI 1.56–15.34), *p*=0.006]. Meanwhile, registered SCFHs were 7.5 times more likely to have good food safety and hygiene practices compared to unregistered SCFHs [aOR=7.50 (95% CI 4.27–13.19), *p*<0.001]. The SCFHs who had completed a food safety training course were 6 times more likely to have good food safety and hygiene practices compared to those who had no such training [aOR=5.97 (95% CI 3.50–10.18), *p*<0.001] (Table 5).

**Table 4:** Association between food safety knowledge, attitude, and hygiene practice and respondent’s socio-demographic characteristics (*n* = 407)

	Food safety knowledge				Food safety attitude				Food safety hygiene practices			
Variable	Good = 274	Poor = 133	*χ*^2^	*p* value	Good = 237	Poor = 170	*χ*^2^	*p* value	Good = 256	Poor = 151	*χ*^2^	*p* value
	*n* (%)	*n* (%)			*n* (%)	*n* (%)			*n* (%)	*n* (%)		
Gender			0.00	0.980			0.57	0.451			0.40	0.525
Male	43 (15.7)	21 (15.8)			40 (16.9)	24 (14.1)			38 (14.8)	26 (17.2)		
Female	231 (84.3)	112 (84.2)			197 (83.1)	146 (85.9)			218 (85.2)	125 (82.8)		
Age group			3.38	0.336			0.63	0.890			1.65	0.648
< 26 years	33 (12.0)	23 (17.3)			32 (13.5)	24 (14.1)			38 (14.8)	18 (11.9)		
26–35 years	110 (40.1)	43 (32.3)			90 (38.0)	63 (37.1)			99 (38.7)	54 (35.8)		
36–45 years	91 (33.2)	46 (34.6)			82 (34.6)	55 (32.4)			81 (31.6)	56 (37.1)		
> 45 years	40 (14.6)	21 (15.8)			33 (13.9)	28 (16.5)			38 (14.8)	23 (15.2)		
Marital status			0.84	0.657			0.35	0.840			1.40	0.496
Single	54 (19.7)	28 (21.1)			49 (20.7)	33 (19.4)			56 (21.9)	26 (17.2)		
Married	212 (77.4)	99 (74.4)			179 (75.5)	132 (77.6)			192 (75.0)	119 (78.8)		
Separated	8 (2.9)	6 (4.5)			9 (3.8)	5 (2.9)			8 (3.1)	6 (4.0)		
Educational status			3.58	0.311			2.88	0.411			15.60	**0.001**
None	45 (16.4)	31 (23.3)			43 (18.1)	33 (19.4)			46 (18.0)	30 (19.9)		
Primary	79 (28.8)	40 (30.1)			74 (31.2)	45 (26.5)			92 (35.9)	27 (17.9)		
Secondary	103 (37.6)	41 (30.8)			86 (36.3)	58 (34.1)			81 (31.6)	63 (41.7)		
Tertiary	47 (17.2)	21 (15.8)			34 (14.3)	34 (20.0)			37 (14.5)	31 (20.5)		
Average monthly income			1.35	0.716			2.95	0.399			16.40	**0.001**
< Ghc500	28 (10.2)	15 (11.3)			25 (10.5)	18 (10.6)			32 (12.5)	11 (7.3)		
Ghc501–1000	115 (42.0)	53 (39.8)			100 (42.2)	68 (40.0)			120 (46.9)	48 (31.8)		
Ghc1001–1500	67 (24.5)	28 (21.1)			60 (25.3)	35 (20.6)			53 (20.7)	42 (27.8)		
> Ghc1500	64 (23.4)	37 (27.8)			52 (21.9)	49 (28.8)			51 (19.9)	50 (33.1)		
Work experience			3.53	0.317			3.99	0.263			2.54	0.467
< 3 years	60 (21.9)	34 (25.6)			53 (22.4)	41 (24.1)			60 (23.4)	34 (22.5)		
3–10 years	143 (52.2)	74 (55.6)			120 (50.6)	97 (57.1)			131 (51.2)	86 (57.0)		
11–20 years	56 (20.4)	22 (16.5)			51 (21.5)	27 (15.9)			51 (19.9)	27 (17.9)		
> 20 years	15 (5.5)	3 (2.3)			13 (5.5)	5 (2.9)			14 (5.5)	4 (2.6)		
Street-cooked food hander registered			4.64	**0.031**			1.31	0.253			42.42	**<0.001**
Yes	209 (76.3)	88 (66.2)			178 (75.1)	119 (70.0)			215 (84.0)	82 (54.3)		
No	65 (23.7)	45 (33.8)			59 (24.9)	51 (30.0)			41 (16.0)	69 (45.7)		
Food safety training course			1.37	0.241			3.50	0.062			36.96	**<0.001**
Yes	115 (42.0)	64 (48.1)			95 (40.1)	84 (49.4)			142 (55.5)	37 (24.5)		
No	159 (58.0)	69 (51.9)			142 (59.9)	86 (50.6)			114 (44.5)	114 (75.5)		

Data presented as the frequency with the corresponding percentage in parenthesis and *p* is significant at < 0.05

**Table 5:** Bivariable and multivariable logistic regression of factors associated with knowledge and hygiene practices on food safety of the respondents (*n* = 407)

	Food safety knowledge				Food safety hygiene practices			
Variable	Good = 274	Poor = 133	cOR (95% CI), *p* value	aOR (95% CI), *p* value	Good = 256	Poor = 151	cOR (95% CI), *p* value	aOR (95% CI), *p* value
	*n* (%)	*n* (%)			*n* (%)	*n* (%)		
Educational status								
None					46 (18.0)	30 (19.9)	1	1
Primary			**–**	**–**	92 (35.9)	27 (17.9)	0.45 (0.24**–**0.84), 0.013**	1.73 (0.51**–**5.95), 0.381
Secondary			**–**	**–**	81 (31.6)	63 (41.7)	1.19 (0.68**–**2.10), 0.542	4.06 (1.63**–**10.11), 0.003**
Tertiary			**–**	**–**	37 (14.5)	31 (20.5)	1.28 (0.66**–**2.49), 0.459	0.82 (0.31**–**2.21), 0.698
Average monthly income								
< Ghc500					32 (12.5)	11 (7.3)	1	1
Ghc501**–**1000			**–**	**–**	120 (46.9)	48 (31.8)	1.16 (0.54**–**2.49), 0.697	0.59 (0.26**–**1.35), 0.214
Ghc1001**–**1500			**–**	**–**	53 (20.7)	42 (27.8)	2.31 (1.04**–**5.11), 0.040*	Empty
> Ghc1500			**–**	**–**	51 (19.9)	50 (33.1)	2.85 (1.30**–**6.27), 0.009**	4.89 (1.56**–**15.34), 0.006**
Street-cooked food handler registered								
Yes	209 (76.3)	88 (66.2)	1.64 (1.04**–**2.59), 0.032*	1.40 (0.87**–**2.26), 0.164	215 (84.0)	82 (54.3)	4.41 (2.78**–**7.01), **<**0.001***	7.50 (4.27**–**13.19), <0.001***
No	65 (23.7)	45 (33.8)	1	1	41 (16.0)	69 (45.7)	1	1
Food safety training course								
Yes			**–**	**–**	142 (55.5)	37 (24.5)	3.84 (2.46**–**5.99), <0.001***	5.97 (3.50**–**10.18), 0.001***
No					114 (44.5)	114 (75.5)	1	1

Significant at ∗*p*< 0.05; ∗∗*p*< 0.01; ∗∗∗*p*< 0.001; *CI* confidence interval, *cOR* crude odds ratio, *aOR* adjusted odds ratio and 1 is the reference

### Pearson correlation between knowledge, attitude, and hygiene practice toward food safety

The study revealed a positive correlation in the knowledge with the attitude outcomes sections (FSA) of food safety (*r*=0.153, *p*=0.002) (Table 6).

**Table 6:** Pearson correlation between knowledge, attitude, and hygiene practice toward food safety

Level	Pearson’s rho	Sig. (2-tailed)
FSK-FSA	0.153**	0.002
FSK-FSHP	0.072	0.146
FSA-FSHP	0.071	0.150

**Correlation is significant at the *p* < 0.01 level (2-tailed); *FSK* food safety knowledge, *FSA* food safety attitude, *FSHP* food safety hygiene practice, *r* Pearson’s rho

## Discussion

The present study investigated knowledge, attitude, and hygiene practices of food safety on SCFHs in North Dayi District of Volta Region, Ghana. This study showed that the majority of SCFHs had good knowledge of food safety. This would help decrease the threat to contamination of foods, food poisoning, and FBDs to the consumers. Studies conducted in Saudi Arabia, Ethiopia, and Ghana have identified the importance of knowledge of food safety to SCFHs and have recommended training programmes on food safety to cultivate the knowledge into hygiene practices [14, 27, 34]. Our finding is inconsistent with previous studies done in Ethiopia and Jordan [38, 45], however consistent with studies conducted in Ghana and Malaysia [47, 54]. The possible reasons could be the type of food training courses received, the sample size, the scoring rubric applied, and understandings acquired on the subjects. This supported claims, creating an optimistic culture of food safety, inhibit food contamination if incorporated periodically [44, 46]. This scenario affirms that the food safety training courses may remarkably enhance the knowledge of food handlers, especially concerning FBDs.

This study found that most of SCFHs knew about the washing of hands for 1 min using liquid and cleanser before touching food, which coincides with the study done in Iran [39]. The washing of hands with soap and water could reduce contamination of hands, cooking utensils, and cooking preparation surfaces leading to a substantive reduction of the risk of FBDs. Our finding does not corroborate with finding from a study done in Malaysia where a vast majority of SCFHs were knowledgeable of the 4th WHO Five Keys to Safer Food to keep the meal at healthy temperatures [20]. In our study, the SCFHs wrongly answered that fresh meat should be bestowed at any place in the fridge once it is cool. This misapplication of temperature could result in contamination and possibly proliferating of microbes in food. The reason is that appropriate temperatures can significantly lessen the risk at which foods will deteriorate, thereby preventing FBDs; hence for safety, foods must be held at an appropriate temperature sufficient to slow down the growth of microorganisms or kill microbes.

Attitude is one of the key elements that influence food safety and the practice and lessen the recurrence of food-related illnesses [51]. This study showed that most of SCFHs had a good attitude toward food safety. It means they understood their roles in food safety which was transmitted into attitude because they possibly serve as a vector for infectious pathogens which lead to food contamination. This agrees with studies conducted in Ghana and Haiti [48, 55], but differs from a study done in Malaysia [36], where the majority of SCFHs had a poor attitude toward food safety. Possibly these could be due to the variances in socio-demographic characteristics, study population, and the study settings. These attitudinal variations could also be due to public reputation preference. Our study showed that visual checking was one of the key ways of differentiating healthy food from rotten ones, which concurs with a study conducted in Iran [39]. This finding is disturbing because the process of identifying food contamination cannot be performed by visual checking, since pathogens or toxins might be present in those foods without necessarily affecting SCFHs’ sensory aspects (smell, colour, or taste); therefore, food handlers who rely on visual checking for the identification of food contamination might expose consumers to an increased risk of contracting FBDs [39, 56]. Therefore, the regulatory authorities must ensure that all SCFHs are trained professionally and certified.

The present study revealed a vast majority of SCFHs agreed that putting uncooked and prepared meal separating prevent cross-contamination, which corresponds to a study done in Haiti [55]. This act of putting fresh foods separating from cooked food could help prevent cross-contamination, which in turn may prevent infections from happening and halt FBDs. This is one of the highly endorsed public health measures to prevent cross-contamination [57]. This study found that almost all of SCFHs agreed that they coughed or sneezed into their elbows if a towel or paper is not available. Coughing and sneezing into the elbow or covering coughs and sneezes, and immediately washing the hands, could help to avert the spread of severe respiratory infections such as influenza and whooping cough. Our finding contradicts with other studies conducted in Malaysia and America; they reported that almost all respondents sneezed right away into their hands and never clean it [20, 58]. This unpleasant attitude is harmful to the public since sneezing and coughing let out droplets of watery and perhaps transmittable microorganisms which can contaminate foods leading to FBDs.

Preservation of good sanitary behaviours is one of the goals for any food establishment, thereby its observance is vital to ensure safe meals for consumers [28, 59]. The proportion of SCFHs in this current study with good hygiene practices of food safety corroborates with previous studies conducted in Saudi Arabia and Ghana [21, 34]. This is an indication that SCFHs can be relied upon to act as the first-line responder to prevent several FBDs when they practice what they know. This would help reduce accidental contamination of foodstuffs due to improper management of cooking utensils and surroundings. Contradictory, in the present study, the scores obtained on the practices section were higher than hygiene practices of food safety reported in studies done in China and Nigeria [25, 60]. The likely explanations of the difference reported could be as a result of the research population, the study cut-off used, the disparity in food safety courses, and differences in the law enforcement regimes. Our study revealed that the level of hygiene practices score was greater than the level of the attitude score attained by the SCFHs which corresponds to a study conducted in Malaysia [15]. The probable justification could be the SCFHs tend to provide responses they trust will create a good picture of their hygiene practices which account for the greater level score. The current study revealed that a vast majority of SCFHs washed their cooking utensils used to cook meals before using them for different meals, which is in line with a study done in Iran [39]. This act is acceptable because food handlers have been mostly identified as a significant vector for food contamination and responsible for FBDs [14, 15]. Our study found that SCFHs practised wrongly by using similar kitchen cloth to clean shells and hands at the time which concurs with a study done in Malaysia [20]. The possible justification could be due to the non-compliance of the respondents to food safety training received. It could also be that they lack understandings of food safety education received. Hence, this displeasing practice may eventually result in contamination of hands and transfers of microorganisms to the consumers. This study showed that a vast majority of SCFHs cleaned fresh food that needs no cooking before consumption, which is in line with a study conducted in Malaysia [20]. This good hygiene practice is necessary to the elementary control of the spread of possibly FBDs.

Our study revealed a positive relationship between knowledge and the attitude of food safety which corresponds to earlier studies conducted in Malaysia, Iran, and Ghana [15, 39, 47]. Nevertheless, the strength of the correlation identified in the knowledge with the attitude scores of food safety was not strong, which implies that it is vital for the respective agency to monitor SCFH activities and enforce safety standards. Previous studies conducted in Malaysia and Iran found no significant relationship in the knowledge with the hygiene practices of food safety [20, 39], which corresponds to our finding but contradicts with studies done in Malaysia and Ghana [15, 47]. This result confirms the assertion that good knowledge does not affect the hygiene performance of food safety [61]. Hence, food handlers should be encouraged by food safety regulatory agencies to at least practise good hygiene irrespective of their levels of knowledge of food safety. In our study, no statistical association was found in the attitudes with the hygiene practice scores of food safety, which opposes earlier studies conducted in Malaysia, Iran, and Ghana [39, 47, 54]. These disparities could be due to their levels of knowledge of food safety and also possibly as a result of the kind of food safety training courses received. This present study found that registered SCFHs were more likely to have good food safety knowledge likened to unregistered SCFHs which is in line with earlier research in Lebanon [51] but differs in the study done in Malaysia [62]. The potential explanation is that maybe before SCFHs have been given their certification of registration, they probably have been taken through food safety training courses which provide them with adequate knowledge of food safety and offer them a good understanding of food poisoning, contamination, and hygiene. This shows the importance of registering food handlers who have successfully been through food safety training courses to acquire knowledge on food safety.

This study showed that the odds of good hygiene practices were higher among SCFHs who had secondary education likened to those with no formal education which is in line with a study conducted in Ethiopia [12]. In contrast to our findings, other studies conducted in Ethiopia and Ghana found SCFHs with primary education as more likely to have good hygiene practices of food safety likened to secondary education [27, 34]. The possible reasons are because most food preparation skills, personal hygiene, and cleanliness are learned from friends, relatives, parents, and media but not necessarily from formal education. However, a lower level of education reduces awareness but the higher one gets educated the better the knowledge which affects their attitude and eventually may reflect into hygiene practices. It implies that food handlers should be encouraged to attain at least basic education before engaging into the cooking business, although it serves as the first sources of income for most uneducated people in the societies. Nevertheless, a study conducted in Ghana showed that regardless of educational background, the food safety actions of SCFHs remain an issue in many nations [48].

The present study showed that SCFHs who earned average monthly income above GHc1500 were more likely to have good hygiene practices compared to respondents who earned less than Ghc500. Our finding confirms a study conducted in Ethiopia and Jordan that found good hygiene practice among food handlers with higher monthly income than those with lower higher monthly income [27, 63]. The possible justification is that SCFHs with high monthly income can afford to purchase items needed to establish themselves in hygienic environments and afford more employees to help in cleaning and waste treatment which could result in a reduction in food poisoning and cross-contamination. This means the high monthly income of food handlers determine their ways of hygiene practices, purchasing more cooking utensils for preparing different meals and managing their leftovers foods to prevent contamination.

The present study showed that registered SCFHs were in favour of good hygiene practices of food safety than the unregistered. The likely description is because of the food safety training courses they received before being registered as food handlers which provides them with an in-depth and comprehensive understanding of hygiene practices such as proper handling of food, personal cleanliness, and sanitation while preparing food. However, there is no research found relating registration of food handlers with hygiene practice scores; hence, the lack of the associated literature offers difficulties to compare our finding to collective results reasonably with concrete answered questions. Nonetheless, our finding shows the importance of registering food handlers after they have been through food safety training courses to encourage them to practise good hygiene.

This study found that SCFHs who have completed training courses on food safety were in favour of good hygiene practices of food safety likened to respondents who had not. Our finding asserts with previous studies done in Ethiopia, Malaysia, and Ghana [36, 38, 47]. The probable justification is that SCFHs who have completed food safety training courses had gained the talents and awareness necessary to handle food safely and sustain great ethics of self-cleanness and hygiene practices. Our finding affirms the assertion that training upsurges understanding of food safety which might reflect into hygiene practices [48]. Hence, a lack of or inadequate training of SCFHs on food safety may inadvertently result in poor hygiene practices, thereby encouraging food contamination [26, 36]. This implies providing food safety training to food handles is important to keep consumers from food poisoning and other wellbeing dangers that could arise from eating unsafe food.

In this present study, it is significant to highpoint SCFHs’ knowledge, attitudes, and hygiene practices are unpredictable from the study conceded, though most of SCFHs properly responded by answering appropriately to related questions of WHO’s Five Keys to Safe Foods guidelines for food handlers. This theoretic-based assessment of the KAP method applied to assessed food handlers’ food safety KAP has some limitations. Firstly, the postulation that the received knowledge on food safety is translated into attitude is not entirely true. The existence of a social desirability bias could similarly have added to the discrepancy amid interview-responded KAP of SCFHs. Social desirability bias is the propensity of SCFHs to provide publically anticipated answers which will be regarded approvingly by people [64]. This proclivity has been shown by their descriptions and overrating socially anticipated KAP questions on food safety. Secondly, as we beforehand mentioned, the research assistants revealed their identities and the purpose of the study to the SCFHs; therefore, the SCFHs were mindful of the hygiene practices and the significance of observing them, but they remained keen to acknowledge their nonconformity and these could likely affect the self-reported hygiene practices. Thirdly, the unavailability of sufficient data from related studies in the district impedes an evaluative comparison of our findings to determine an improvement of food safety KAP among SCFHs; therefore, our findings ought to be interpreted with caution. However, due to the representative nature of the sample assessed, the findings of this study can be generalized to other SCFHs in the district. After all, it makes a substantial impact concerning food safety KAP in North Dayi District because it is the first study conducted in the district that presents an imperative foundation for design to increase food safety and hygiene practice in the district, region, and beyond.

## Conclusion

Over half of the respondents had good levels of KAP of food safety. This study found a significant relationship in the knowledge and hygiene practice scores of food safety with SCFH registration. This shows the importance of strict enforcement of registration and certification of SCFHs by regulatory agencies as a means of protecting the consuming public. Therefore, the government agency through FDA should intensify the vitality of undertaking food safety training on WHO’s Five Keys to Safer Food by food handlers before being registered. Furthermore, the District Health Directorate should properly and effectively supervise food handlers engaging in cooking businesses to ensure they transmit the link between knowledge with the attitude of food safety into hygiene practice. Further studies should assess the kind of food safety training modules received and their impacts on the KAP of WHO’s Five Keys to Safer Foods as well as evaluating their hygiene practices with observational checklists.

## Supplementary Information

**Additional file 1.**

## Data Availability

The datasets generated during and/or analyzed during the current study are not publicly available due to ethical consideration but are available from the corresponding author on reasonable request.
